# Single-cell DNA and RNA sequencing of circulating tumor cells

**DOI:** 10.1038/s41598-021-02165-7

**Published:** 2021-11-24

**Authors:** Masato Kojima, Takanori Harada, Takahiro Fukazawa, Sho Kurihara, Isamu Saeki, Shinya Takahashi, Eiso Hiyama

**Affiliations:** 1 Graduate School of Biomedical and Health Sciences, Hiroshima University, Hiroshima, Japan; 2 Department of Pediatric Surgery, Hiroshima University Hospital, Hiroshima, Japan; 3 Natural Science Center for Basic Research and Development (N-BARD), Hiroshima University, Hiroshima, Japan

**Keywords:** Molecular biology, Oncology

## Abstract

Single-cell sequencing of circulating tumor cells can precisely represent tumor heterogeneity and provide useful information for cancer treatment and research. After spiking TGW neuroblastoma cells into blood derived from healthy volunteer, the cells were isolated by fluorescence-activated cell sorting. DNA and mRNA were amplified by four different whole-genome amplifications (WGA) and three whole-transcriptome amplifications (WTA) methods, followed by single-cell DNA and RNA sequencing. Multiple displacement amplification (MDA)-based WGA methods showed higher amplification efficiency than other methods with a comparable depth of coverage as the bulk sample. The uniformity of coverage greatly differed among samples (12.5–89.2%), with some samples evaluated by the MDA-based WGA method using phi29 DNA polymerase and random primers showing a high (> 80%) uniformity of coverage. The MDA-based WTA method less effectively amplified mRNA and showed non-specific gene expression patterns. The PCR-based WTA using template switching with locked nucleic acid technology accurately amplified mRNA from a single cell. Taken together, our results present a more reliable and adaptable approach for CTC profiling at the single-cell level. Such molecular information on CTCs derived from clinical patients will promote cancer treatment and research.

## Introduction

Recently, circulating tumor cells (CTCs) have been considered to represent the precise tumor heterogeneity and overall tumor characteristics^1^. In previous studies^2,3^, cellular heterogeneity in CTCs was shown to reflect the spectrum of mutations in tumor tissues, including primary and metastatic sites, and the existence of small subpopulations with different malignant profiles. CTC profiling at the single-cell level most accurately represents tumor heterogeneity and provides useful information for cancer treatment and research.

Because of the low counts of CTCs, they must first be enriched and isolated before determining their profiles. CTC isolation is performed using various technologies based on the biological or physical properties of CTCs, such as fluorescence-activated cell sorting (FACS) and using microfluidics platforms^4^. After isolating the CTCs, their DNA and mRNA is amplified by whole-genome or whole-transcriptome amplification (WGA, WTA, respectively). WGA is classified as PCR-based, isothermal multiple displacement amplification (MDA)-based, and PCR with MDA hybridized methods such as multiple annealing and loop-based amplification cycling (MALBAC)^5^. Similar to WGA, many WTA methods can be used for low-input RNA samples. These methods use different priming strategies (polyT priming or random priming with ribosomal RNA depletion), second strand cDNA synthesis (polyA tailing or template switching), and cDNA amplification (PCR or isothermal in vitro transcription) approaches^6,7^. Variations in these methods can affect amplification efficiency and accuracy, as well as introduce unwanted bias. In this study, we simulated single-cell sequencing of CTCs and used a TGW neuroblastoma cell line expressing high levels of the specific cell surface marker GD2^8^. After isolating the CTCs by FACS, single-cell DNA and RNA were amplified by various WGA and WTA methods. We compared PCR-based, MDA-based, and PCR with MDA hybridized methods in WGA and there are only few studies compared PCR- and MDA-based cDNA amplification, so in the present study, we compared PCR-based and MDA-based methods in WTA. Then, we evaluated the performance of single-cell sequencing by next-generation sequencing (NGS).

## Results

### Experimental design

TGW neuroblastoma cells were spiked into blood derived from a healthy volunteer for comparison of WGA and WTA (around 0.1% of the peripheral mononuclear cell fractions). These cells were isolated by FACS using an anti-GD2 antibody. We examined four different WGA methods as follows. In the PCR-based method, named as method A, fragmented DNA after enzymatic digestion is ligated to linker adaptors with universal sequences and amplified by linker adaptor-specific primers^5^. The MDA-based method, named as method B, uses the DNA primase *Thermus thermophilus* (Tth) PrimPol without artificial primers. TthPrimPol randomly synthesizes short DNA primers, and phi29 DNA polymerase begins processive polymerization using these primers^9^. Another MDA-based method, named as method C, uses artificial random primers for processive polymerization by phi29 DNA polymerase^9^. The PCR with MDA hybridized method, named as method D, generates looped DNA molecules by eight cycles of multiple displacement preamplification using specifically designed MALBAC primers and Bst DNA polymerase with strand displacement activity. The looped amplicons are further amplified by PCR^10^.

The quality check (QC) of the WGA samples was performed by multiplex qPCR of eight cancer-related genes, *BRAF*, *EGFR*, *KIT*, *KRAS*, *NRAS*, *PIK3CA*, *PTEN*, and *TP53*, as previously described for the optimization of CTC DNA sequencing^11^. QC criteria was set as threshold cycles (Ct values) ≤ 30 in all genes. We next evaluated the depth and uniformity of coverage using Hiseq 2500 (Illumina) for comprehensive genetic profiling of CTCs at the single-cell level.

**Figure 1:**
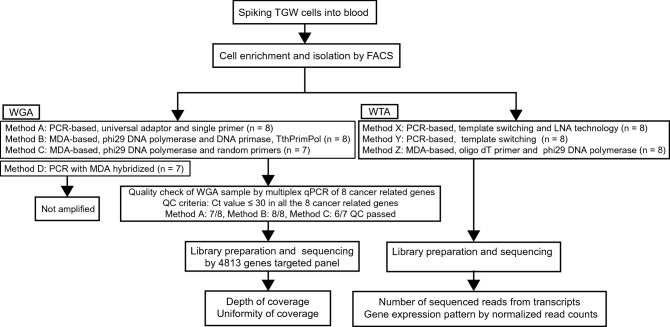
Experimental design. TGW neuroblastoma cells were spiked into blood derived from a healthy volunteer and isolated by fluorescence-activated cell sorting (FACS) using anti-GD2 and CD90 antibodies. We amplified DNA and mRNA from isolated single TGW cells using several different WGA and WTA methods. We performed a quality check (QC) of the WGA samples by multiplex qPCR of eight cancer-related genes, and QC criteria was set as threshold cycles (Ct values) ≤ 30 in all the genes. Next, we performed single-cell sequencing and evaluated the depth and uniformity of coverage for WGA samples, the number of sequenced reads from transcripts and gene expression patterns for WTA samples. *FACS* fluorescence-activated cell sorting, *WGA* whole-genome amplification, *WTA* whole-transcriptome amplification, *PCR* polymerase chain reaction, *MDA* multiple displacement amplification, *LNA* locked nucleic acid technology.

We examined three different WTA methods consisting of two PCR-based methods and one MDA-based method. The PCR-based methods, named as methods X and Y, use oligo-dT primers to reverse-transcribe polyA mRNA and template switching for second-strand cDNA synthesis. Method X uses locked nucleic acid (LNA) technology with template-switching oligonucleotides containing modified guanosine and locks the first-strand cDNA, contributing to efficient second-strand cDNA synthesis^12^. The MDA-based method, named as method Z, uses oligo-dT primers to reverse-transcribe polyA mRNA and phi29 DNA polymerase for cDNA amplification. We evaluated the number of sequenced reads from transcripts and gene expression patterns using Hiseq 2500 and Miseq for comprehensive transcriptomic profiling at the single-cell level. We also compared these parameters between WGA, WTA samples, and the bulk samples of 1 × 10^6^ TGW cells (Fig. 1). A schematic overview of the mechanisms of the WGA and WTA methods used in this study is shown in Fig. S1 and S2.

### Amount of WGA sample

The amounts of DNA after WGA were 1.6 ± 0.2 µg (mean ± standard deviation (SD), n = 8) by method A (PCR-based), 1.9 ± 0.3 µg (mean ± SD, n = 8) by method B (MDA-based), 20.5 ± 5.3 µg (mean ± SD, n = 7) by method C (MDA-based), and 0.17 ± 0.004 µg (mean ± SD, n = 7) by method D (PCR with MDA hybridized).

**Figure 2:**
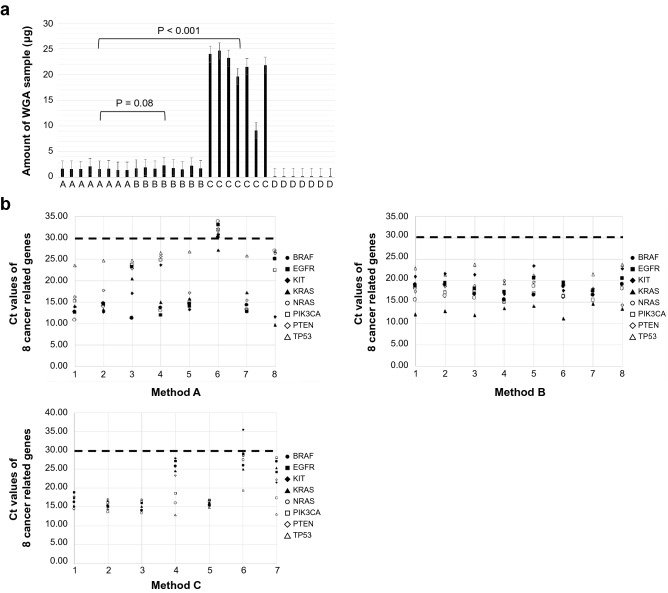
Amount and quality of WGA sample. (**a**) Amounts of WGA samples obtained by each method. Method C (MDA-based) showed high productivity, but the amounts of WGA samples by method D (PCR with MDA hybridized) were too low to be evaluated by multiplex qPCR for QC. (**b**) Ct values of 8 cancer related genes, *BRAF*, *EGFR*, *KIT*, *KRAS*, *NRAS*, *PIK3CA*, *PTEN*, *TP53* in WGA samples by each method. QC criteria was set as threshold cycles (Ct values) ≤ 30 in all 8 cancer related genes. In method A (PCR-based), seven of eight samples passed QC, in method B (MDA-based), all eight samples passed QC, and in method C (MDA-based), six of seven samples passed QC. In WGA samples assumed as low-quality, the Ct values of the 8 genes and their deviations were high.

Method C (MDA-based) showed high productivity, but the amounts of WGA samples obtained by method D (PCR with MDA hybridized) were too low to be evaluated by multiplex qPCR for QC (Fig. 2a).

### Quality of WGA sample

Seven of eight samples passed QC in method A (PCR-based), all eight samples passed QC in method B (MDA-based), and six of seven samples passed QC in method C (MDA-based). The Ct values of the eight genes and their deviation were increased in WGA samples, indicating low quality (Fig. 2b). The Ct values of the eight cancer-related genes in each WGA sample are shown in Table S2.

### Performance of NGS in WGA sample

In the bulk sample, the total number of sequenced reads were 15.0 × 10^6^, depth of coverage was 46.4, and uniformity of coverage was 94.8%. In the QC passed WGA samples (n = 7) by method A (PCR-based), the number of total sequenced reads was 4.5 × 10^6^ ± 1.2 × 10^6^ (mean ± SD), depth of coverage was 8.3 ± 1.9 (mean ± SD) and uniformity of coverage was 40.1. ± 16.7% (mean ± SD). In the QC passed WGA samples (n = 8) by method B (MDA-based), the numbers of total sequenced reads were 18 × 10^6^ ± 5.0 × 10^6^ (mean ± SD), depth of coverage was 45.7 ± 15.2 (mean ± SD) and uniformity of coverage was 32.8. ± 14.5% (mean ± SD). In the QC passed WGA samples (n = 6) by method C (MDA-based), the number of total sequenced reads was 21 × 10^6^ ± 4.5 × 10^6^ (mean ± SD), depth of coverage was 68.9 ± 18.8 (mean ± SD) and uniformity of coverage was 71.5 ± 24.6% (mean ± SD).

**Figure 3:**
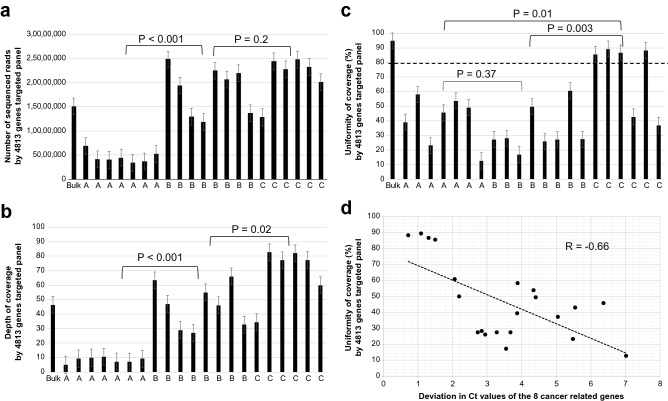
Performance of NGS in WGA sample. (**a**) Numbers of sequenced reads in the bulk sample and WGA samples by each method. Method A (PCR-based) yielded a substantially smaller number of sequenced reads compared to methods B and C (MDA-based). Method B and method C (MDA-based) yielded a comparable number of sequenced reads as the bulk sample. (**b**) Depth of coverages in the bulk sample and WGA samples by each method. Consistent with the number of sequenced reads, methods B and C (MDA-based) yielded a comparable depth of coverage as the bulk sample, and method A (PCR-based) yielded a substantially lower depth of coverage. (**c**) Uniformity of coverages in the bulk sample and WGA samples by each method. Uniformity of coverage was defined as the percentage of sequenced base position with the depth of coverage greater than 0.2 × the mean depth of coverage. Uniformity of coverage greatly differs among WGA samples despite passing the QC and only samples of method C (MDA-based) showed over 80% uniformity of coverage. (**d**) Uniformity of coverage and the deviation of Ct values of the 8 cancer-related genes. The deviation of Ct values of the 8 genes in qPCR was well-correlated with the uniformity of coverage in NGS (R = 0.66) and the low deviation of Ct values of the 8 genes contributed to a high uniformity of coverage.

Method A (PCR-based) yielded a substantially smaller number of sequenced reads compared to methods B and C (MDA-based), and methods B and C (MDA-based) yielded a comparable number of sequenced reads as the bulk sample. Consistent with the number of sequenced reads, methods B and C (MDA-based) yielded a comparable depth of coverage with the bulk sample, and method A (PCR-based) showed a substantially lower depth of coverage (Fig. 3a,b).

The uniformity of coverage in the bulk sample was over 90%; in contrast, the uniformity of coverage significantly differed among WGA samples (12.5–89.2%) despite passing QC. Method C (MDA-based) yielded higher uniformity of coverage than method A (PCR-based) and method B (MDA-based), and method C showed over 80% uniformity of coverage (Fig. 3c). The deviation in Ct values of the eight genes in qPCR correlated well with the uniformity of coverage in NGS (R = −0.66), and the low deviation of Ct values of the eight genes contributed to a high uniformity of coverage (Fig. 3d).

### Performance of NGS in WTA sample

In the bulk samples (× 3 technical replicates), the number of sequenced reads from transcripts was 30.6 × 10^6^ and number of expressed genes was 12,175. In WTA samples (n = 8) by method X (PCR-based), the number of sequenced reads from transcripts was 2.8 × 10^6^ ± 0.4 × 10^6^ (mean ± SD) and number of expressed genes was 5283 ± 392 (mean ± SD). In WTA samples (n = 8) by method Y (PCR-based), the number of sequenced reads from transcripts was 2.3 × 10^6^ ± 0.2 × 10^6^ (mean ± SD) and number of expressed genes was 3426 ± 513 (mean ± SD). In WTA samples (n = 8) by method Z (MDA-based), the number of sequenced reads from transcripts was 1.0 × 10^6^ ± 0.5 × 10^6^ (mean ± SD) and number of expressed genes was 3006 ± 1357 (mean ± SD).

**Figure 4:**
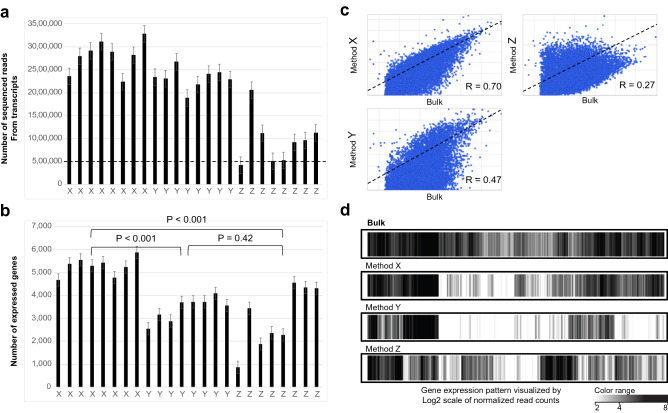
Performance of NGS in WTA sample. (**a**) Numbers of sequenced reads from transcripts in WTA samples by each method. The number of sequenced reads by method Z (MDA-based) was much lower than those of the other methods (PCR-based). However, most expressed genes in single cell can be detected with 0.5 × 10^6^ reads and almost all samples yielded over 0.5 × 10^6^ reads from transcripts. (**b**) Numbers of expressed genes in WTA samples by each method. We defined transcripts with over 100 sequenced reads as expressed genes. The number of expressed genes was comparable between each sample, and method X (PCR-based) amplified higher number of expressed genes compared to the other methods. (**c**) Scatter plot of normalized read counts of expressed genes in the bulk sample and WTA samples by each method. Sequenced reads from transcripts were normalized by DESeq and correlation analysis of the gene expression patterns between the bulk sample and WTA samples was performed by Spearman’s test. The samples by method X (PCR-based) correlated well with the bulk sample compared to the other methods (R = 0.70). (**d**) Gene expression patterns in the bulk sample and WTA samples by each method. According to scatter plot, the gene expression patterns of the samples by method X resembled those of the bulk sample. In contrast, samples by method Z (MDA-based) showed non-specific gene expression patterns.

The number of sequenced reads from transcripts by method Z (MDA-based) was significantly lower than that of the other methods (PCR-based) (Fig. 4a). Most expressed genes in a single cell can be detected with 0.5 × 10^6^ reads, and almost all samples yielded over 0.5 × 10^6^ reads from the transcripts^13^. Consistent with the previous study, the number of expressed genes was comparable between each sample, and method X (PCR-based) amplified a larger number of expressed genes to some extent compared to the other methods (Fig. 4b).

We performed correlation analysis of the gene expression patterns between the bulk sample and WTA samples by each method and obtained correlation coefficients of 0.70 (method X, PCR-based), 0.47 (method Y, PCR-based), and 0.26 (method Z, MDA-based) (Fig. 4c). The heatmap of gene expression patterns showed that WTA samples by method X (PCR-based) closely resembled the bulk sample, whereas WTA samples by method Z (MDA-based) exhibited non-specific gene expression patterns (Fig. 4d). In WTA samples by method Z (MDA-based), genes showing high expression in the bulk sample were expressed at low levels; whereas those with low expression levels in the bulk sample were highly expressed in WTA samples.

## Discussion

It is known that PCR-based and PCR with MDA-hybridized WGA methods generate high amplification uniformity, whereas the MDA-based WGA method shows high amplification efficiency but generates amplification bias through high processive amplification by phi29 DNA polymerase, contributing to lower amplification uniformity^5^. In this study, MDA-based WGA methods showed higher amplification efficiency than the PCR-based WGA method and produced a comparable depth of coverage with the bulk sample, but the uniformity of coverage varied widely among samples. As a QC criteria, we used Ct values ≤ 30 in all eight cancer-related genes by multiplex qPCR according to a previous study^11^; however, this criteria was not adequate for selecting samples with high uniformity of coverage. The deviation in Ct values of the eight genes correlated well with the uniformity of coverage, and a low deviation may be useful for QC evaluation of WGA samples. Interestingly, in the samples by the PCR-based WGA method, the Ct values showed very high deviation, but the uniformity of coverage was not as low. A low depth of coverage contributed to the reduced uniformity of coverage, but the high deviation in Ct values may have been caused by enzymatic digestion before adaptor ligation to DNA fragments in this workflow. Site-specific DNA digestion may result in fewer amplification products and DNA fragments that are too small or large^5^, and performing QC by multiplex qPCR may not be suitable for such a PCR-based WGA method. We also compared two different MDA-based WGA methods. Both high processive amplification by phi29 DNA polymerase and the priming inequality of random primers can cause amplification bias^9^; therefore, we compared the combinations of phi29 DNA polymerase and random primers or DNA primase, TthPrimPol. Consistent with a previous study^14^, TthPrimPol showed low amplification uniformity, and the REPLI-g Advanced DNA Single Cell Kit (method C) using a combination of phi29 DNA polymerase and random primers showing higher uniformity. The MDA-based WGA method using a combination of high-fidelity phi29 DNA polymerase and random primers along with stricter QC criteria by multiplex qPCR may show high performance for comprehensive genetic profiling of CTCs at the single-cell level.

In contrast to the WGA method, the MDA-based WTA method showed lower amplification efficiency than the PCR-based WTA methods. A read count of 0.5 × 10^6^ from transcripts is thought to cover most expressed genes in a single cell, and the number of expressed genes in WTA samples by each method was not as different as the number of sequenced reads. However, the MDA-based WTA method produced non-specific gene expression patterns, which possibly occur because of amplification bias to shorter or fragmented low-quality mRNA caused by phi29 polymerase and linear cDNA amplification. CEL-seq using the T7 promoter and linear amplification by in vitro transcription is similar to the MDA-based WTA method and accurately quantified mRNA levels using unique molecular identifiers (UMIs) in a previous study^15^. UMIs are stretches of 4–10 random nucleotides integrated into sequencing primers and serve as a random barcode for each mRNA molecule^16^. By counting each UMI only once rather than counting the total sequenced reads, amplification bias can be eliminated^16^. If we use the MDA-based WTA method, the integration of UMIs such as CEL-seq may improve the accuracy of the gene expression data by reducing amplification bias. In contrast, the SMART-Seq HT Kit (method X, PCR-based) showed higher amplification efficiency than the other methods, and the gene expression pattern was well-correlated with that in the bulk sample. The SMART-Seq HT Kit (method X) and method Y were built on the same SMART-seq (switching mechanism at the 5′ end of the RNA transcript) technique. The differences between these methods are that only the SMART-Seq HT kit uses LNA technology and the hands-on time is shorter than method Y and LNA technology may contribute to efficient and accurate cDNA synthesis and amplification.

In conclusion, we performed single-cell sequencing of CTCs using a combination of FACS and various WGA and WTA methods. The MDA-based WGA method using the combination of phi29 DNA polymerase and random primers showed high performance for single-cell DNA sequencing, whereas the PCR-based WTA method using the combination of template switching and LNA technology showed high performance for single-cell RNA sequencing. Although we present a more reliable and adaptable approach for CTC profiling at the single-cell level, further investigation of other metrics such as the accuracy of variant calling or tools of QC of WTA samples to perform single-cell sequencing of CTCs derived from clinical patients should be performed. Such molecular information on CTCs derived from clinical patients will promote cancer treatment and research.

## Methods

### Extraction of DNA and RNA from bulk of TGW cells

The human neuroblastoma cell line, TGW, was obtained from the American Type Culture Collection (Manassas VA, USA) and maintained as recommended previously^17^. DNA and RNA were extracted from 1 × 10^6^ TGW cells using DNA Extractor WB Kit (FUJIFILM Wako Pure Chemical Corporation, Osaka, Japan) and miRNeasy Mini kit (Qiagen, Hilden, Germany) according to the manufacturer’s protocols.

### Spiking TGW cells into blood and cell enrichment and isolation by FACS

We spiked 1 × 10^4^ TGW cells into 7.0 mL whole blood samples derived from a healthy volunteer. Cell enrichment was performed by density gradient centrifugation using Ficoll-PaquePlus separation medium (Greiner Bio-On, Kremsmunster, Austria). We used phycoerythrin-labeled anti-GD2 antibody (BD Biosciences, Franklin Lakes, NJ, USA), allophycocyanin-labeled anti-CD90 antibody (BioLegend, San Diego, CA, USA), fluorescein isothiocyanate-labeled anti-CD45 antibody (BioLegend), PerCP-Cy5.5 labeled anti-CD235a antibody (BioLegend), and DAPI (Takara Bio, Shiga, Japan) for multiparametric FACS. Finally, we isolated GD2^+^ and CD90^+^, CD45^-^, CD235a^-^, and DAPI^-^ cells using FACS Aria II (BD Biosciences). We randomly selected single TGW cells for each WGA, WTA method.

### WGA

Ampli1 WGA Kit (Silicon Biosystems, Castel Maggiore, Italy), named as method A, is a PCR-based method using a linker adaptor with a universal sequence and single primer. TruePrime Single Cell WGA Kit (Sygnis, Heidelberg, Germany), named as method B, is an MDA-based method using phi29 DNA polymerase and DNA primase, TthPrimPol, rather than artificial primers. REPLI-g Advanced DNA Single Cell Kit (Qiagen), named as method C, is an MDA-based method using phi29 DNA polymerase and random primers. MALBAC Single Cell WGA Kit (Yikon Genomics, Jiangsu, China), named as method D, is a PCR with MDA hybridized method, and generates looped DNA molecules via eight cycles of multiple displacement preamplification using specifically designed MALBAC primers and Bst DNA polymerase. The looped amplicons are further amplified by PCR^10^. The WGA product concentration was measured using Qubit fluorometer 3.0 (Thermo Fisher Scientific, Waltham, MA, USA). Experimental procedures were performed by well-disciplined technicians according to the manufactures’ instructions.

### QC of WGA sample

The QC of WGA samples was performed by multiplex qPCR of eight cancer-related genes, *BRAF*, *EGFR*, *KIT*, *KRAS*, *NRAS*, *PIK3CA*, *PTEN*, and *TP53*, as described previously for the optimization of CTC DNA sequencing^11^. Pre-amplification was performed using ProFlex™ PCR System (Applied Biosystems, Foster City, CA, USA) with multiplex PCR Kit (Qiagen) using 80 ng of DNA per sample according to the previous study^11^. Multiplex qPCR was performed using CFX-96 real-time PCR detection system (Bio-Rad, Hercules, CA, USA) with Brilliant III Ultra-Fast SYBR® Green QPCR Master Mix (Agilent Technologies, Santa Clara, CA, USA). PCR products were assessed by Ct values, and WGA samples with Ct values ≤ 30 in all eight cancer-related genes were further evaluated.

### WTA

SMART-Seq HT Kit (Takara Bio), named as method X, is a PCR-based method that uses oligo-dT primers and template switching. LNA technology is used in this method for efficient cDNA synthesis by template switching oligonucleotides containing modified guanosine and locks the first-strand cDNA. NEBNext Single Cell/Low Input RNA Library Prep Kit (New England Biolabs, Ipswich, MA, USA), named as method Y, is a PCR-based method that uses oligo-dT primers and template switching only. QIAseq FX Single Cell RNA Library Kit (Qiagen), named as method Z, is an MDA-based method that uses oligo-dT primers and phi29 DNA polymerase. Experimental procedures were performed by well-disciplined technicians according to the manufactures’ instructions.

### Library preparation and sequencing

Libraries for DNA sequencing were prepared using TruSight One Sequencing Panel (Illumina, San Diego, CA, USA) targeting 4813 genes. The libraries of the bulk sample and WGA samples by each method were equally pooled at a final loading concentration of 8 pM and paired-end 150 bp sequencing was performed by Hiseq 2500 (Illumina).

Libraries for RNA sequencing of the bulk sample were prepared using TruSeq Stranded mRNA Library Prep Kit (Illumina). Library preparation for RNA sequencing of the WTA sample by method X was performed using Nextera XT DNA Library Preparation Kit (Illumina) according to the manufacturer’s instructions. The libraries of the bulk sample and WTA samples by methods X and Z were equally pooled at a final loading concentration of 8.5 pM and paired-end 75 bp sequencing was performed by Hiseq 2500 (Illumina). The libraries of WTA samples by method Y were equally pooled at a final loading concentration of 12 pM and paired-end 75 bp sequencing was performed by Miseq (Illumina).

### Data analysis

FASTQ files of DNA sequencing were imported into CLC Genomics Workbench version 11.0.1 (CLC Bio, Aarhus, Denmark) and sequenced reads were aligned to the UCSC hg19 reference genome. The uniformity of coverage was calculated as the percentage of sequenced base positions in which the depth of coverage was greater than 0.2 × the mean depth of coverage^18^.

FASTQ files of RNA sequencing were processed using Strand NGS version 2.7 (Strand Life Sciences, Bangalore, India), and sequenced reads were aligned to the UCSC hg19 reference genome. We selected reads aligned to exonic regions and defined transcripts with over 100 sequenced reads as the expressed genes in each sample.

### Statistical analysis

Student t-test was used to compare each WGA, WTA method and statistical significance was defined as P < 0.05. The correlation coefficient between uniformity of coverage and deviation in the Ct values of the eight genes in QC was determined by Pearson’s test. In RNA sequencing, sequenced reads from transcripts were normalized by DESeq, and correlation analysis of the gene expression patterns between the bulk sample and WTA samples was performed by Spearman’s test. Other data were reported as the mean ± SD, and analyses were performed using Microsoft Excel software.

## Supplementary Information

Supplementary Legends.

Supplementary Figure S1.

Supplementary Figure S2.

Supplementary Tables.

## Data Availability

The multiplex qPCR data analyzed in this study are included in this published article and supplementary materials. The sequencing data analyzed in this study are not publicly available but can be obtained from the corresponding author.
