# Supplementary material for: Single-cell DNA and RNA sequencing of circulating tumor cells
Source: Sci Rep. 2021 Nov 24;11:22864. doi: 10.1038/s41598-021-02165-7 (PMC8613180; doi:10.1038/s41598-021-02165-7)
Supplement: Supplementary file 1 — Supplementary Legends. [file 41598_2021_2165_MOESM1_ESM.docx]

**Supplementary Figure legends**

**Supplementary Figure S1.** **Overview of mechanisms of WGA methods in this study.**

Method A is a PCR-based method. DNA was fragmented by incubation with MseI restriction endonuclease for 5 min at 37 °C. Fragmented DNAs were ligated to linker adaptors (orange lines) with universal sequences (21 bp) and amplified by 44 cycles of PCR using linker adaptor specific primers (blue lines).

Method B is an MDA-based method involving isothermal amplification by phi29 DNA polymerase (blue circles) with strand displacement activity. Phi29 DNA polymerase possesses high amplification processivity up to 10 kb in length but contributes to a lower uniformity of coverage than PCR-based method. This method uses the DNA primase *Thermus thermophilus* (Tth) PrimPol (red circles) rather than random primers to remove the priming inequality of random primers and produce a higher uniformity of coverage. TthPrimPol randomly synthesizes short DNA primers (red lines), and phi29 DNA polymerase performs processive polymerization using these primers. Isothermal amplification was performed using a mixture of TthPrimPol and phi29 DNA polymerase for 3 h at 30 °C.

Method C is also an MDA-based method using random primers (red lines) rather than DNA primase TthPrimPol of method B. Isothermal amplification was performed using a mixture of random primers and phi29 DNA polymerase (blue circles) for 2 h at 30 °C.

Method D is a PCR with MDA hybridized method which generated looped DNA molecules by eight cycles of multiple displacement preamplification for 1 h. MDA-based preamplification is performed using specifically designed MALBAC primers (red and blue lines) with complementary sequences (27 bp) and Bst DNA polymerase (light blue circles) with strand displacement activity, and complementary sequences form looped amplicons. Loop formation prevents the amplicons from being used as templates for multiple displacement preamplification. The looped amplicons were further amplified by 17 cycles of PCR.

WGA, whole-genome amplification; PCR, polymerase chain reaction; MDA, multiple displacement amplification

**Supplementary Figure S2.** **Overview of mechanisms of WTA methods in this study.**

Few comparative studies of PCR and MDA-based cDNA amplification have been performed; therefore, we examined three different WTA methods consisting of two PCR-based methods and one MDA-based method.

Methods X and Y are PCR-based methods and based on the same SMART-seq (Switching Mechanism at the 5′ end of the RNA Transcript) technique. Reverse transcription is performed using oligo-dT primers (orange lines) and Moloney murine leukemia virus reverse transcriptase with terminal transferase activity. When reverse transcription reaches the 5′ end of the RNA template, a few untemplated nucleotides, mainly 2–5 cytosines, are added to the 3′ end of the newly synthesized first cDNA strand. These extra nucleotides function as a docking site for TSOs that carry three riboguanosines and specific sequences (23 bp in method X, 22 bp in method Y) for PCR amplification. Reverse transcription proceeds using TSOs as a template and finishes the synthesis of first cDNA strand, and cDNA amplification is performed in 19 cycles of PCR. Method X uses locked nucleic acid technology with TSOs containing modified guanosine (+G) and locks the first cDNA strand, contributing to efficient cDNA synthesis and amplification.

Method Z is an MDA-based method. Reverse transcription is performed using oligo-dT primers and Quant Reverse Transcriptase. cDNA was amplified by isothermal MDA using phi29 DNA polymerase (blue circles) for 2 h at 30 °C.

WTA, whole-transcriptome amplification; PCR, polymerase chain reaction; MDA, multiple displacement amplification; TSOs, template switching oligonucleotides; LNA, locked nucleic acid technology
