# Supplementary material for: Single-cell DNA and RNA sequencing of circulating tumor cells
Source: Sci Rep. 2021 Nov 24;11:22864. doi: 10.1038/s41598-021-02165-7 (PMC8613180; doi:10.1038/s41598-021-02165-7)
Supplement: Supplementary file 2 — Supplementary Figure S1. [file 41598_2021_2165_MOESM2_ESM.pdf]

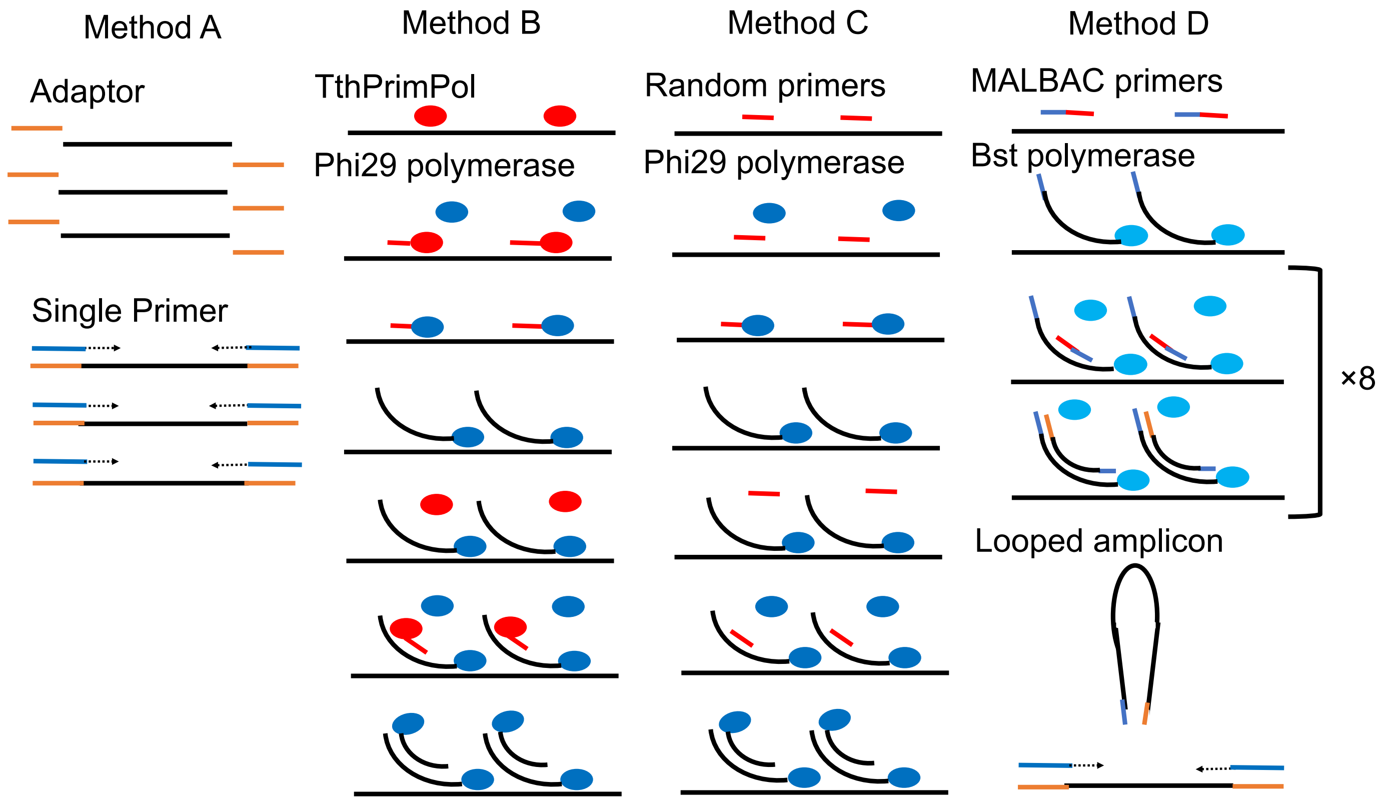

Method A
Adaptor
Single Primer
Method B
TthPrimPol
Phi29 polymerase
Method C
Random primers
Phi29 polymerase
Method D
MALBAC primers
Bst polymerase
×8
Looped amplicon
