# Supplementary material for: Single-cell DNA and RNA sequencing of circulating tumor cells
Source: Sci Rep. 2021 Nov 24;11:22864. doi: 10.1038/s41598-021-02165-7 (PMC8613180; doi:10.1038/s41598-021-02165-7)
Supplement: Supplementary file 3 — Supplementary Figure S2. [file 41598_2021_2165_MOESM3_ESM.pdf]

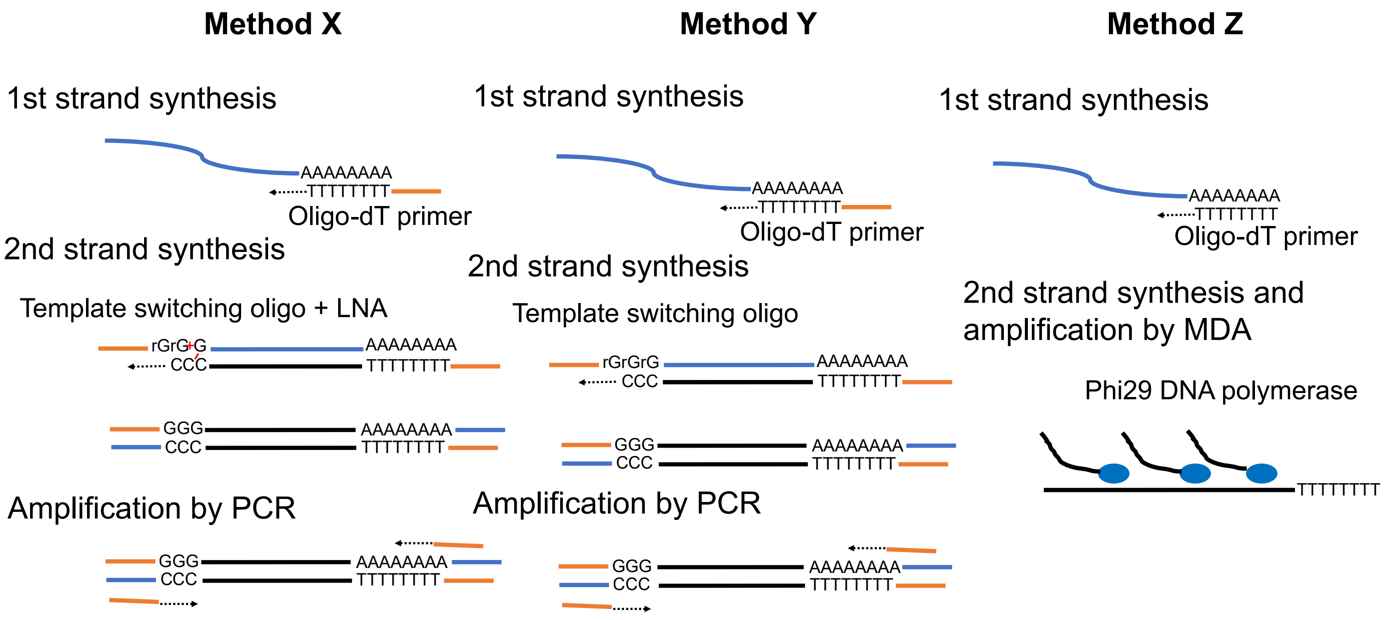
Method X
Method Y
Method Z
1st strand synthesis
AAAAAAAA
TTTTTTTT
Oligo-dT primer
2nd strand synthesis
Template switching oligo + LNA
rGrG+G
CCC
GGG
Amplification by PCR
Template switching oligo
rGrGrG
2nd strand synthesis and amplification by MDA
Phi29 DNA polymerase
